# Liquiritin Alleviates Pain Through Inhibiting CXCL1/CXCR2 Signaling Pathway in Bone Cancer Pain Rat

**DOI:** 10.3389/fphar.2020.00436

**Published:** 2020-04-24

**Authors:** Huadong Ni, Miao Xu, Keyue Xie, Yong Fei, Housheng Deng, Qiuli He, Tingting Wang, Songlei Liu, Jianjun Zhu, Longsheng Xu, Ming Yao

**Affiliations:** Department of Anesthesiology and Pain Research Center, The Affiliated Hospital of Jiaxing University, Jiaxing, China

**Keywords:** CXCL1, CXCR2, liquiritin, glia-neuron interaction, bone cancer pain, spinal cord

## Abstract

Bone cancer pain (BCP) is an intractable clinical problem, and lacked effective drugs for treating it. Recent research showed that several chemokines in the spinal cord are involved in the pathogenesis of BCP. In this study, the antinociceptive effects of liquiritin, which is an active component extracted from Glycyrrhizae Radix, were tested and the underlying mechanisms targeting spinal dorsal horn (SDH) were investigated. The BCP group displayed a significant decrease in the mechanical withdrawal threshold on days 6, 12, and 18 when compared with sham groups. Intrathecal administration of different doses of liquiritin alleviated mechanical allodynia in BCP rats. The results of immunofluorescent staining and western blotting showed that liquiritin inhibited BCP-induced activation of astrocytes in the spinal cord. Moreover, intrathecal administration of liquiritin effectively inhibited the activation of CXCL1/CXCR2 signaling pathway and production of IL-1β and IL-17 in BCP rats. In astroglial-enriched cultures, Lipopolysaccharides (LPS) elicited the release of chemokine CXCL1, and the release was decreased in a dose-dependent manner by liquiritin. In primary neurons, liquiritin indirectly reduced the increase of CXCR2 by astroglial-enriched-conditioned medium but not directly on the CXCR2 target site. These results suggested that liquiritin effectively attenuated BCP in rats by inhibiting the activation of spinal astrocytic CXCL1 and neuronal CXCR2 pathway. These findings provided evidence regarding the the antinociceptive effect of liquiritin on BCP.

## Background

Bone cancer pain (BCP) is often severe and intractable, and has a strong effect on the quality of life of cancer patients. But no breakthrough has been achieved yet with regard to the therapeutic strategies and mechanisms of BCP (Falk and Dickenson, 2014; van den Beuken-van Everdingen et al., 2018). Therefore, novel and more efficacious therapies are imperative to improve the quality life of patients. Research studies over the past decade have suggested that proinflammatory cytokines such as Interleukin-1 beta (IL-1β) and tumor necrosis factor alpha (TNF-α) from glial cells (astrocytes and microglia) are responsible for BCP and might augment nociceptive signals in the spinal cord (Chiang et al., 2012; Lu et al., 2015). The activated glial cells release the proinflammatory cytokines that act on their receptors and express on postsynaptic neurons, leading to post-synaptic hyperexcitability and facilitatory pain transmission (Guo et al., 2007; Jin et al., 2018; Wang et al., 2018).

CXCL1 belongs to the CXC family, and promote both nociceptor and central sensitization through its primary receptor CXCR2, which in turn is regarded as a promising target for novel analgesic drugs (Silva et al., 2017). Under varied pathological states, activated astrocytes are considered as the main source of CXCL1 (Zhang Z. J. et al., 2017). Recent findings strongly suggested that CXCL1 might act on CXCR2 *via* glial-neuronal interactions in the spinal cord in several pathological pain models (Cao et al., 2014; Chen et al., 2014). In addition to this, our recent study also demonstrated that CXCL1-CXCR2 signaling plays a critical role in glial-neuron interactions and in descending facilitation of BCP (Ni et al., 2019a).

**Figure 1:**
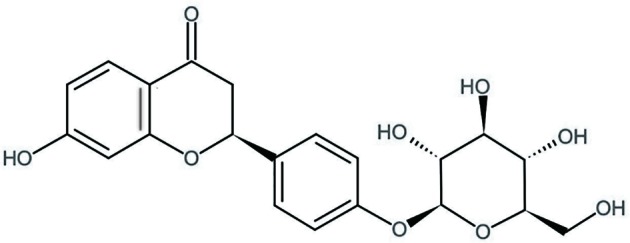
The chemical structure of Liquiritin (LQ, C_21_H_22_O_9_). The chemical class is flavonoids.

Glycyrrhiza uralensis Fisch is used as a traditional Chinese herb from long time and is commonly used to treat injuries or swelling due to its association with wide range of pharmacological effects. Liquiritin (LQ) (**Figure 1**) is one of the major constituents of Glycyrrhizae Radix, which has a great potential in medical applications and pharmacological activities, such as in preventing inflammation and relieving pain, cancer, cough, and allergic reactions (Zhang and Ye, 2009; Xie et al., 2017; Li et al., 2018). Zhai et al. have reported that LQ ameliorates rheumatoid arthritis (RA) by reducing proinflammatory cytokines (IL-6) and blocking MAPK signalling (Zhai et al., 2019). *In vivo* studies have revealed that LQ is used for effective treatment of neuropathic pain by down-regulating the cytokines including TNF-α and IL-6, suggesting it as a promising anti-inflammatory and anti-nociceptive drug (Zhang M. T. et al., 2017). However, the role of LQ in BCP alleviation has not been reported yet, and the underlying mechanisms of anti-nociceptive effects of LQ on BCP require further elucidation.

Hence, in the present study, the possible antinociceptive effects of LQ and its effects on glial activation were evaluated by utilizing the BCP model. Considering the inhibitory effects of LQ on inflammatory action, the expressions of several pro-inflammatory cytokines as well as CXCL1 after LQ administration were investigated. These results assisted in supporting the potential clinical application of LQ in preventing BCP.

## Methods

### Animals

All experiments were approved by the Animal Care and Use Committee of Jiaxing University (Jiaxing, China). Adult female Sprague-Dawley rats weighing 180–220 g were obtained from the Experimental Animal Center of Jiaxing University. All rats were housed at a constant room temperature of (23°C ± 1°C) on a 12-h light/dark cycle with free access to food and water. All efforts were made to minimize animal suffering and number of animals used.

### Modeling of BCP Rats

The BCP model has been established as described in the previous study (Ni et al., 2016). In brief, the animals were first anesthetized by pentobarbital sodium [50 mg/kg, intraperitoneally (i.p.)]. A hole was then carefully drilled into the right tibia for inoculation. Walker 256 cells (1 × 10^6^ cells/10 μl) or heat-killed cells (sham group) were cautiously injected into the bone medullary canal. After that, the cells were allowed to fill the bone cavity for 2 min. The syringe was withdrawn and the injection site was immediately closed with bone wax to prevent the leakage of the cells at the injection site.

### Drugs and Administration

LQ (purity≥98.0%) was purchased from Beijing Zhongke Quality Inspection Biotechnology Co., Ltd. (lot no. 551-15-5) and was suspended in sodium carboxymethyl cellulose (0.5% CMC-Na). Next, sodium pentobarbital (Sigma, Germany) was dissolved in saline solution (0.9% NaCl), and then was injected i.p., whereas different concentrations of LQ (20, 100, 500, and 1,000 μg/kg, once daily) were injected intrathecally (i.t.) for 7 days and were freshly prepared prior to conducting the experiments.

### Rotarod Testing

Motor dysfunction demonstrated evident effects in nociceptive behavioral tests. In order to assess whether intrathecal administration of LQ influenced motor function, the rats underwent rotarod tests by an accelerating rotating rod (UgoBasile, Varese, Italy). All animals were habituated for 3 min of training daily for at least 5 days before baseline testing. The rats were then placed on the rotarod, with a diameter of 70 mm, which was then linearly accelerated from 4 to 40 rpm within a course of 3 min time. The rat was placed on the rotating rod, and the time it stayed on the rod within a cut-off time of 3 min was measured. The final results are expressed as the percentage of baseline value of each group.

### Mechanical Allodynia Test

Mechanical allodynia was tested by using a set of Von Frey monofilaments (BME-404, Institute of Biological Medicine, Academy of Medical Science, China) according to our previous report (Ni et al., 2019b). Firstly, all the animals were allowed to habituate to the testing environment daily for at least 2 days before undergoing baseline testing. The rats were then placed on a wire-mesh floor covered with a plexiglass chamber (20×10×10 cm) and allowed it for at least 30 min for habituation. The monofilaments were held against the plantar surface of the hind paw until the rats withdrew their paw or licked their feet. After five consecutive tests with 10 s intervals, the average values were taken to record and were defined as paw withdrawal thresholds (PWT). PWT was measured in rats prior to undergoing surgery and on days 3, 6, 12, or 18 post-surgery, as well as at various time points after intrathecal injection.

### Primary Cell Culture

Primary astrocytes were cultured as described previously (Qian et al., 2015). In brief, cerebral cortices of neonatal rat brains were removed and triturated, followed by filtering through a 100 μm nylon screen. All cells were seeded into 75 cm^2^ flasks containing 10% FBS, penicillin (100 U/ml), and streptomycin (100 μg/ml) in high glucose DMEM. After culturing for 2 weeks, the astrocytes were prepared by shaking the flasks for 3 h and then incubated with 10 ml of 0.05% trypsin in an incubator for 15 min to separate the microglia and oligodendrocytes from the astrocytes. The prepared astrocytes exhibited a purity of 80%–95% as determined by glial fibrillary acidic protein (GFAP) immunoreactivity, which showed a star-shaped morphology with the processes extending from the soma.

Prior to stimulation with LPS, the DMEM medium was replaced by Opti-MEM medium. The cells were then incubated with LPS for different time periods ranging from 0.5 h to 6 h. LQ (1, 10, 100 μM) or vehicle was added into the medium 3 h after LPS treatment. After these treatments, the cells were collected for western blotting (WB) and real-time polymerase chain reaction (RT-PCR) analysis.

The cerebral cortices were harvested from the neonatal rat brains (within 24 h), and immersed in the digestive fluid. The cell suspension was planted into the plates pre-coated with cell adherent reagent. After incubation for 6 h in DMEM with 10% FBS, the medium was then changed to neurobasal medium containing B27 supplement and 0.5 mM glutamine. All the experiments were initiated 5–6 days after planting. The harvested neurons showed dendritic spine morphology and exhibited about 90% purity according to NeuN immunoreactivity. LQ (1, 10, 100 μM) and SB225002 (10 μM) treatments were initiated at 3 h and 60 min after LPS treatment (10 μg/ml, 60 min at 37°C), respectively. Astroglial-enriched-conditioned medium pre-treated with LQ (1, 10, 100 μM) was used as a substitute for half of the neuronal medium.

### Cell Viability Analysis

The viability of astroglial-enriched cultures was assessed by MTT assay. In brief, the astroglial-enriched cultures were seeded into 96-well plates at a cell density of 4 × 10^4^ cells/well. After that, the cells were stimulated with varied concentrations of LQ (0,10, 25, 50, 75, 100, 150, and 200 μM) with or without LPS (10 μg/ml) stimulation for 24 h followed by addition of 20 μl of MTT (5 mg/ml) and then incubation at 37°C for 4 h. The supernatant was discarded after centrifugation and DMSO (200 μl) was added followed by oscillation for 15 min to form crystal dissolution prior to measuring the absorbance at 570 nm (Thermo, Waltham, MA, USA).

### Real-Time Quantitative PCR

Total RNA of L4-5 spinal segmental tissue or cultured cells was extracted using Trizol reagent (Invitrogen, Carlsbad, USA). One microgram of total RNA was reverse transcribed using a mixture of random primers according to the manufacturer’s protocol (Takara, Shiga, Japan). RT-PCR analysis was performed using the Real-time Detection System (Rotor-Gene 6000, Hamburg, Germany) by a SYBR Green PCR kit (Takara). The cDNA was amplified using the following primers: CXCL1 forward, 5′-GGCAGGGATTCACTTCAAGA-3′; CXCL1 reverse, 5′-ATCTTGAGCTCGGCAGTGTT -3′, CXCR2 forward, 5′-CGTTCTGGTGACTTTGCTGA-3′; CXCR2 reverse, 5′-ACAGAGCAGGTGCTTCGATT-3′; glyceraldehyde-3-phosphate dehydrogenase (GAPDH)-forward (5′-AAATGGTGAAGGTCGGTGTGAAC-3′), and GAPDH-reverse (5′-CAACAATCTCCACTTTGCCACTG-3′). The PCR amplifications were performed at 95°C for 30 s, followed by 40 cycles of thermal cycling at 95°C for 5 s and 60°C for 45 s. Data were collected after each cycle and displayed graphically (Rotor-Gene 6000 Series Software 1.7). Quantification was performed by normalizing the cycle threshold (Ct) values with GAPDH Ct and analyzed using the 2^−△△Ct^ method.

### Western Blot

The L4-5 spinal segmental tissue or cultured cells was homogenized using RIPA lysis buffer (Millipore) containing a mixture of phosphatase and proteinase inhibitors. The sample was centrifuged at 15,000 rpm for 15 min and then the supernatant was collected. The protein concentrations were detected by BCA protein assay (Pierce). Equal amounts of protein (50 μg) were separated on 10% SDS-PAGE gel and then transferred onto the PVDF membrane. The membrane was blocked with 5% skimmed milk at room temperature (RT) for 2 h and incubated with antibodies CXCL1 (1:400, rabbit, Boster), CXCR2 (1:400, rabbit, Abcam), and GAPDH (rabbit, 1:20,000; Sigma-Aldrich) for overnight at 4°C. This membrane was washed and further incubated with horseradish peroxidase-conjugated goat anti-rabbit IgG (1:30,000, Bioworld) at RT for 2 h. The immunoreactive bands were detected by enhanced chemiluminescence (Thermo Scientific) and exposed to X-ray films. GAPDH was used as an internal control.

### Immunofluorescence

Animals were deeply anesthetized with pentobarbital sodium (50 mg/kg, i.p.). After cardiac perfusion [with 0.01 M phosphate buffered saline (PBS), following 4% paraformaldehyde at 4°C at PH 7.4], the lumbar spinal cord tissues (L4-5) were harvested and fixed in 4% paraformaldehyde at PH 7.4 for 24 h at 4°C, followed by preservation in 30% sucrose for 24 h for subsequent cryoprotection. The coronal sections of spinal cord at 30-μm were collected with a freezing microtome and washed in 0.01 M PBS. The sections were blocked with 5% standard donkey serum and 0.3% Triton X-100 for 1 h at RT, and then incubated with primary antibodies for overnight at 4°C. Subsequently, the sections that were washed in 0.01 M PBS were incubated with secondary antibodies for 2 h at RT. The images of ipsilateral spinal cord were captured using a fluorescence microscope (Leica Microsystems, Wetzlar, Germany).

For determining the specificity of CXCR2 antibody, the antigenic peptide preabsorption/neutralization method was used according to the manufacturer’s instructions. A blocking peptide with antigenic sequence of CXCR2 protein (EDLSNYSYSSTLPPFLLDAAPC) was synthesized (Thermo Fisher Scientific). The CXCR2 antibody was mixed with a fivefold (by weight) excess of the blocking peptide in TBS. The mixture was then incubated for 2 h at room temperature. The normal staining protocol described above was then followed using the CXCR2 antibody only or CXCR2 antibody+blocking peptide. The staining intensities of these two groups were compared thereafter.

The cultured astrocytes and neurons after incubation with LPS combines with the Vehicle or LQ, followed by fixing with 4% paraformaldehyde for 30 min, and disposing for immunofluorescence with CXCL1 antibody (1:100), CXCR2 antibody (1:100), GFAP antibody (1:1,000), and NeuN antibody (1:300), as described above. Finally, DAPI (Bioss) was added for 20 min at 28 ± 2°C for nuclear staining.

### Statistical Analysis

All data were analyzed by SPSS version 20.0 and expressed as means ± SEM. One-way analysis of variance (ANOVA) or two-way repeated measures ANOVA followed by Bonferroni’s were used for comparison among the groups. D’Agostino-Pearson omnibus and Levene’s tests were used to evaluate data distribution and equality of variance analysis. P < 0.05 was considered to be statistically significant.

## Results

### Mechanical Allodynia Induced by Bone Cancer and Effects of Intrathecal Repeated Administration of LQ on Rotarod Test

**Figure 2:**
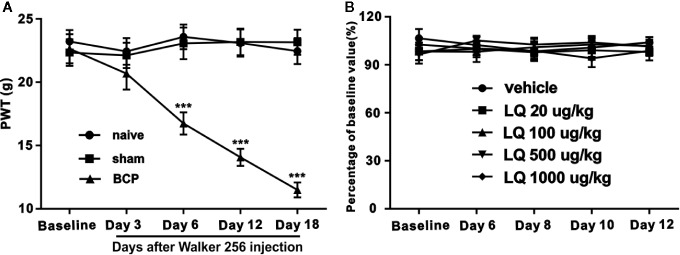
Mechanical allodynia induced by bone cancer and the effects of intrathecally administered Liquiritin (LQ) on motor performance of naïve rats in rotarod test. After operation, the mechanical pain domain has no obvious change in sham group. In contrast, in bone cancer pain (BCP) group, paw withdrawal thresholds (PWT) began to decrease on day 6 until day 18 (F_2,21_ = 22.94, ^***^P < 0.001 vs. control; n = 8, two way ANOVA, **2A**). Compared with baseline response, intrathecal injection of Liquiritin (LQ) (20, 100, 500, and 1,000 ug/kg) for 7 days did not affect the motor performance (F_4,35_ = 0.5979, P > 0.05 vs. control; n = 8, two way ANOVA, **2B**). The results were expressed as percentage of each rat’s own baseline value.

After injection of walker 256 cells, the PWT was measured on days 0, 3, 6, 12, or 18 post-surgery. The mechanical withdrawal threshold showed a significant decrease on day 6 and continued to decline at a certain speed as a sign of successful modeling (F_2,21_ = 22.94, ^***^P < 0.001 vs. control; n = 8, two way ANOVA, **Figure 2A**). In contrast, the sham group rats injected with heat-killed walker 256 cells showed no obvious change on the mechanical withdrawal threshold *(F_2,21_ = 22.94, P > 0.05 vs. naïve; n = 8, two way ANOVA*, **Figure 2A**).

Motor dysfunction demonstrated evident effects on the results of nociceptive behavior and was considered essential for assessing whether the dosages of LQ (20, 100, 500, and 1,000 ug/kg) could induce impairment of motor functions. A rotarod test was performed to assess the influence of LQ by repeated administration on motor function. As shown in **Figure 2B**, no difference was observed in the performance of rats in the vehicle control group or in the LQ treatment (20, 100, 500, and 1,000 ug/kg) group, indicating that repeated intrathecal administration of LQ demonstrated no obvious measurable effect on motor functions *(F_4,35_ = 0.5979, P > 0.05 vs. control; n = 8, two way ANOVA,* **Figure 2B**).

### LQ Ameliorates Bone Cancer-Induced Mechanical Allodynia in a Dose-Dependent Manner

**Figure 3:**
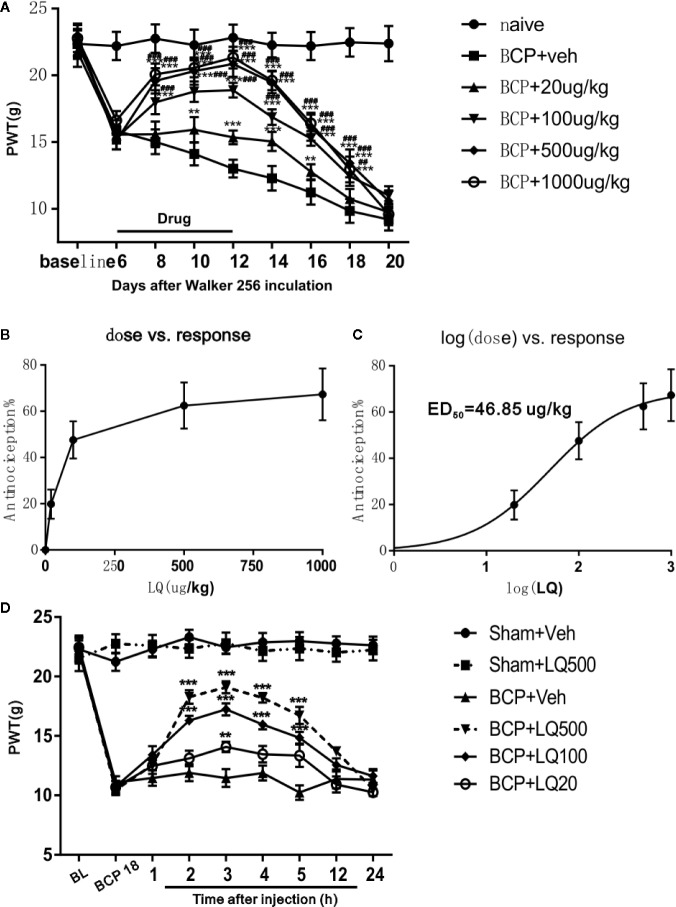
Effects of intrathecal Liquiritin (LQ) administration on bone cancer induced mechanical allodynia. **(A)** Intrathecal injection of LQ dose-dependently alleviated the mechanical allodynia in bone cancer pain (BCP) rats and this effect was still observed 6 days after drug withdrawal on day 18 (F_5,42_ = 21.4, ^**^P < 0.01, ^***^P < 0.001 vs. BCP + Veh group, ^##^P < 0.01, ^###^P < 0.001 vs. BCP + 20 μg/kg group; n = 8, two way ANOVA, **3A**). The dose-effect or log (dose)-effect curves for the analgesic effects of intrathecally administered LQ were shown in **(B**, **C)**. The ED50 of LQ on bone cancer-induced mechanical allodynia was 46.85 μg/kg. **(D)** Intrathecal injection of single-dose of LQ on postoperative day (POD) 18 i.t. still alleviated paw withdrawal thresholds (PWT). The effects reached peak at 3 h after injection and then gradually faded away, where only the 20 ug/kg works at 3 h after injection. (F_5,42_ = 105.8, ^**^P < 0.01, ^***^P < 0.001 vs. BCP + Veh group; n = 8, two way ANOVA).

To assess whether LQ ameliorates BCP, different doses (20, 100, 500, and 1,000 ug/kg) of LQ were i.t. injected once per day for 7 days from postoperative days (POD) 6–12. LQ treatment did not change the basal threshold in the rats of sham-operated group (data not shown). Compared with control group, intrathecal administration of LQ at 100, 500, and 1,000 ug/kg has significantly elevated the PWTs of BCP rats in a dose-dependent manner. Their effect was started on POD 8, i.t., 2 days after beginning LQ treatment, and the analgesic effects of LQ were still observed on POD 18, i.t., 6 days after the treatment was stopped (F_5,42_ = 21.4, ^**^P < 0.01, ^***^P < 0.001 vs. BCP + Veh group, ^##^P < 0.01, ^###^P < 0.001 vs. BCP + 20 μg/kg group; n = 8, two way ANOVA, **Figure 3A**). However, the effect of LQ at low dosage, 20 μg/kg, was started on POD 10 and lasted only for 4 days after drug withdrawal (F_5,42_ = 21.4, ^**^P < 0.01, ^***^P < 0.001 vs. BCP + Veh group; n = 8, two way ANOVA, **Figure 3A**). Compared with control group, the effects of LQ on BCP-induced mechanical allodynia were further calculated based on the log (dose)-response curve (**Figure 3B**). To calculate the median effective dose (ED50), the dose-response curve was also calculated (**Figure 3C**). The ED50 of LQ on bone cancer-induced mechanical allodynia was 46.85 μg/kg (**Figure 3C**). To examine whether the LQ effects the mechanical allodynia in the later period during BCP, single-dose LQ (20, 100, and 500 ug/kg) i.t. were given on POD 18. The effects were observed at 2 h after injection and lasted for 5 h, except for 20 ug/kg, wherein it works only after 3 h of injection (F_5,42_ = 105.8, ^**^P < 0.01, ^***^P < 0.001 vs. BCP + Veh group; n = 8, two way ANOVA, **Figure 3D**).

### LQ Inhibited BCP-Induced Elevated Astrocytic Activation

**Figure 4:**
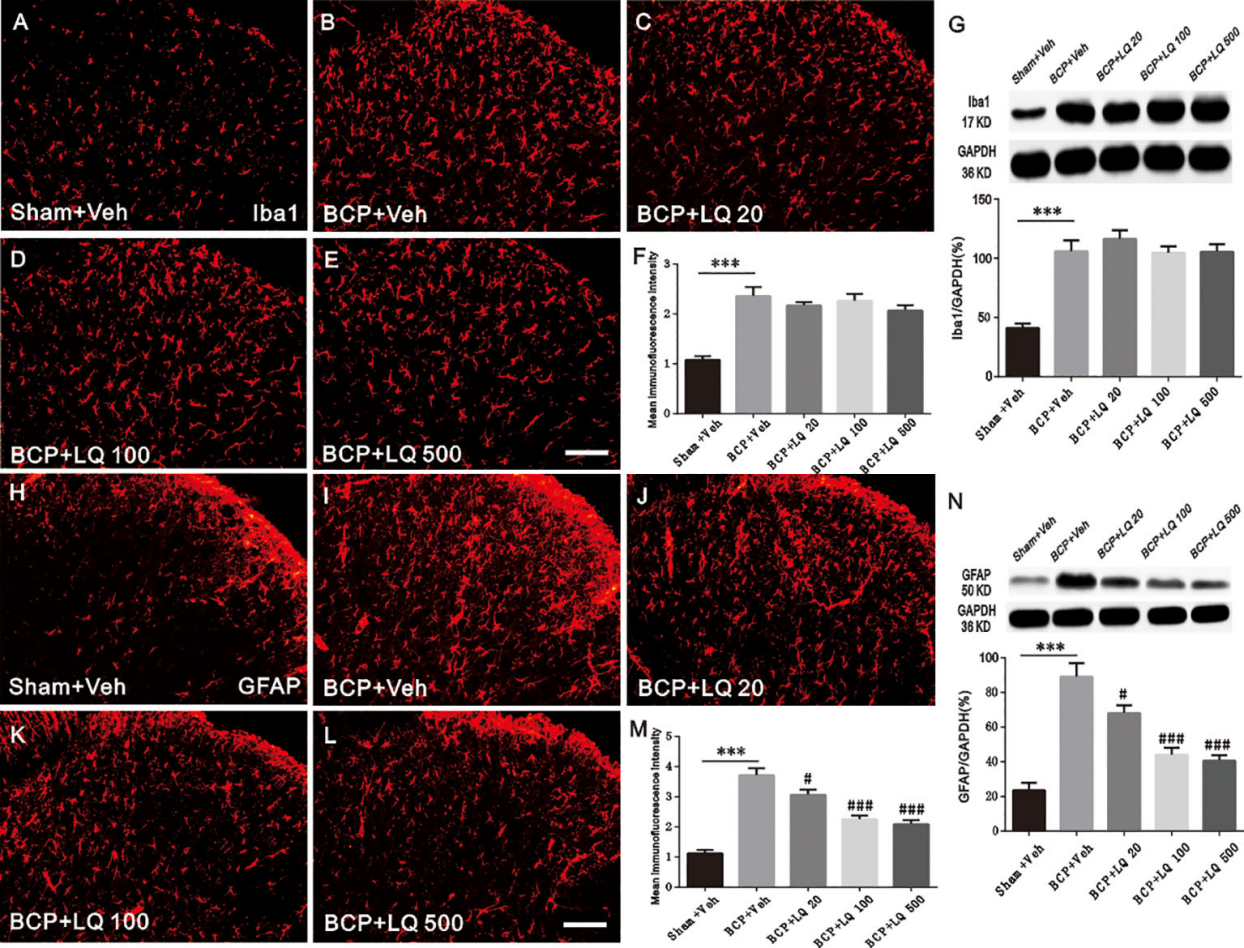
Effect of intrathecal administration Liquiritin (LQ) on glial activation in the spinal dorsal horn of bone cancer pain (BCP) rats on postoperative day (POD) 12. **(A–E)** BCP induced a remarkable microglial activation, which was indicated by Iba1 upregulation in the ipsilateral spinal dorsal horn on POD 12. Intrathecal LQ administration did not inhibit the immunodensities of Iba1 in the ipsilateral spinal dorsal horn after BCP. Scale bar 100 μm; mean immunofluorescence intensity **(F)** and western blot **(G)** of Iba1 expressions after different treatments (F_4,15_ = 18.54, ^***^P < 0.001 vs. sham + Veh group, P > 0.05 vs. BCP + Veh group; F_4,15_ = 19.82, ^***^P < 0.001 vs. sham + Veh group, P > 0.05 vs. BCP + Veh group; n = 4, one way ANOVA, **4F, G**). **(H–L)** BCP induced activation of astrocytes remarkably, which was indicated by GFAP upregulation in the ipsilateral spinal dorsal horn on POD 12. Intrathecal LQ administration inhibited immunodensities of GFAP in the ipsilateral spinal dorsal horn after BCP. Scale bar 100 μm; mean immunofluorescence intensity **(M)** and western blot **(N)** of GFAP expressions after different treatments (F_4,15_ = 37.49, ^***^P < 0.001 vs. sham + Veh group; ^#^P < 0.05, ^###^P < 0.001 vs. BCP + Veh group; F_4,15_ = 25.18, ^***^P < 0.001 vs. sham + Veh group;^#^P < 0.05, ^###^P < 0.001 vs. BCP + Veh group; n = 4, one way ANOVA, **4M, N**).

To verify whether the anti-allodynic effects of LQ are accompanied with the inhibition of glial activation, Iba1 (microglia marker) and GFAP (astrocyte marker) expressions at POD 12 among the groups were investigated. Immunohistochemical data showed that BCP induced Iba1 and GFAP expression in the ipsilateral spinal dorsal horn, especially in the spinal cord laminae I–II, on POD 12 when compared with sham + Veh group (**Figures 4A, B, H, I**). WB and immunoreactive intensity also verified the induction of Iba1 by BCP and GFAP expression (F_4,15_ = 18.54, ^***^P < 0.001 vs. sham + Veh group, P > 0.05 vs. BCP + Veh group; F_4,15_ = 19.82, ^***^P < 0.001 vs. sham + Veh group, P > 0.05 vs. BCP + Veh group; F_4,15_ = 37.49, ^***^P < 0.001 vs. sham + Veh group; ^#^P < 0.05, ^###^P < 0.001 vs. BCP + Veh group; F_4,15_ = 25.18, ^***^P < 0.001 vs. sham + Veh group; ^#^P < 0.05, ^###^P < 0.001 vs. BCP + Veh group; n = 4, one way ANOVA, **Figures 4A–N**). Intrathecal administration of LQ daily from POD 6 to 12 resulted in significantly decreased expression of GFAP in the spinal dorsal horn (**Figures 4H–L**). WB (**Figure 4N**) also demonstrated that the expression of GFAP was significantly attenuated with 20, 100, and 500 ug/kg LQ treatment (F_4,15_ = 25.18, ^***^P < 0.001 vs. sham + Veh group; ^#^P < 0.05, ^###^P < 0.001 vs. BCP + Veh group; n = 4, one way ANOVA, **Figure 4N**). In contrast, intrathecal administration of LQ did not decrease Iba1 immunofluorescence and protein in spinal dorsal horn (F_4,15_ = 18.54, ^***^P < 0.001 vs. sham + Veh group, P > 0.05 vs. BCP + Veh group; F_4,15_ = 19.82, ^***^P < 0.001 vs. sham + Veh group, P > 0.05 vs. BCP + Veh group; n = 4, one way ANOVA, **Figures 4F**). This demonstrated that the proliferation of microglia was unaffected by LQ.

### LQ Inhibited BCP-Induced CXCL1 Upregulation in the Spinal Astrocytes

**Figure 5:**
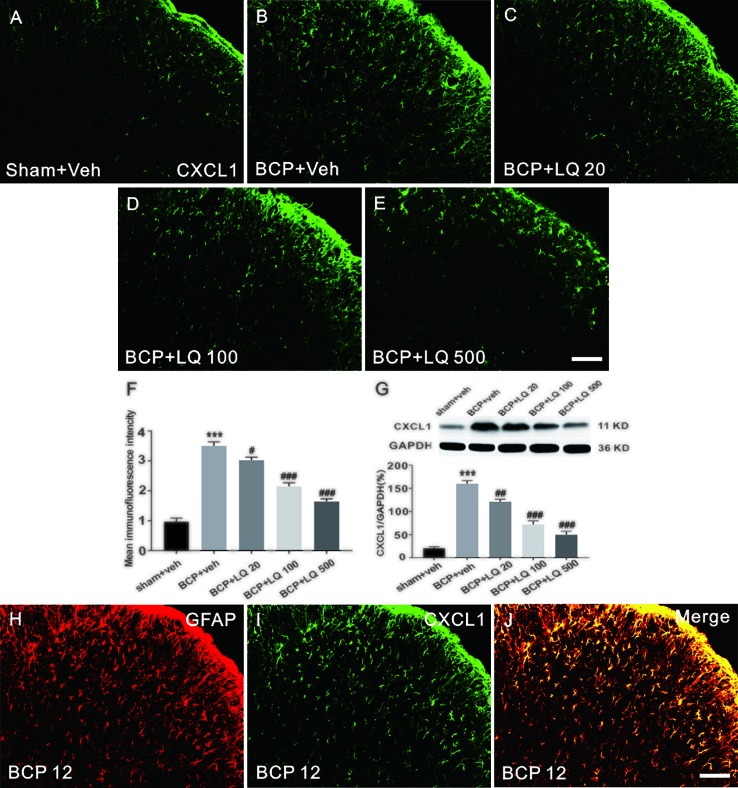
Effects of intrathecal injection of Liquiritin (LQ) on bone cancer pain (BCP)-induced CXCL1 upregulation in spinal astrocytes. **(A–E)** BCP induced a remarkable upregulation of CXCL1 in the ipsilateral spinal dorsal horn. Intrathecal LQ administration inhibited BCP-induced increase in CXCL1 immunoreactivity in the spinal cord. Scale bar 100 μm; mean immunofluorescence intensity **(F)** and western blot **(G)** of CXCL1 expressions after different treatments (F_4,15_ = 82.27, ^***^P < 0.001 vs. sham + Veh group; ^#^P < 0.05,^###^P < 0.001 vs. BCP + Veh group; F_4,15_ = 79.38, ^***^P < 0.001 vs. sham + Veh group; ^##^P < 0.01, ^###^P < 0.001 vs. BCP + Veh group; n = 4, one way ANOVA, **F, G**). **(H–J)** Immunostaining images demonstrated CXCL1(green) was predominantly co-localized with GFAP (red) as shown by overlapped staining (the rightmost panel, yellow). Scale bar, 100 μm.

The expression of CXCL1 in spinal astrocytes after LQ treatment was investigated. The spinal cords on POD 12 were harvested after continuous injection once per day from POD 6 to 12. Immunohistochemical data showed that CXCL1 expression was significantly increased on day 12 in BCP rats (**Figures 5A, B**). Additionally, all the 3 doses of LQ inhibited the increase of BCP-induced CXCL1 immunoreactivity in the spinal cord (**Figures 5A–E**). Statistical analysis of intensity and WB results further showed that LQ inhibited BCP-induced CXCL1 expression on day 12 *(F_4,15_ = 82.27, ^#^P < 0.05, ^###^P < 0.001 vs. BCP + Veh group; F_4,15_ = 79.38, ^##^P < 0.01, ^###^P < 0.001 vs. BCP + Veh group; n = 4, one way ANOVA,* **Figures 5F, G**). Moreover, LQ attenuated BCP-induced increase in CXCL1 protein expression in the spinal cord, with high dose of LQ being the most effective *(F_4,15_ = 82.27, ^#^P < 0.05, ^###^P < 0.001 vs. BCP + Veh group; F_4,15_ = 79.38, ^##^P < 0.01, ^###^P < 0.001 vs. BCP + Veh group; n = 4, one way ANOVA,* **Figures 5F, G**). These results suggested that intrathecal LQ has effectively inhibited CXCL1 upregulation in a dose-dependent manner. Accordingly, the double staining data demonstrated that CXCL1 showed its expression in astrocytes on POD 12 (**Figures 5H–J**).

### LQ Attenuated BCP-Induced CXCR2 Upregulation in Spinal Cord Neurons

**Figure 6:**
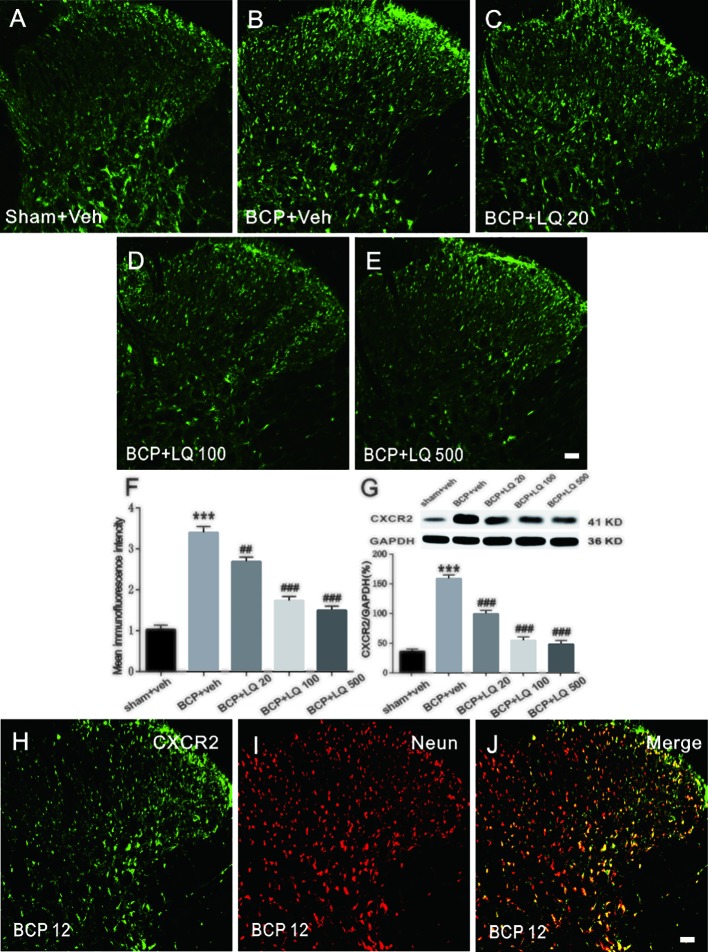
Effects of intrathecal injection of Liquiritin (LQ) on bone cancer pain (BCP)-induced CXCR2 upregulation in the spinal neurons. **(A–E)** BCP induced a remarkable upregulation of CXCR2 in the ipsilateral spinal dorsal horn. Intrathecal LQ administration inhibited BCP-induced increase in CXCR2 immunoreactivity in the spinal cord. Scale bar 100 μm; mean immunofluorescence intensity **(F)** and western blot **(G)** of CXCR2 expressions after different treatments (F_4,15_ = 66.22, ^***^P < 0.001 vs. sham + Veh group; ^##^P < 0.01, ^###^P < 0.001, vs. BCP + Veh group; F_4,15_ = 72.94, ^***^P < 0.001 vs. sham + Veh group; ^###^P < 0.001 vs. BCP + Veh group; n = 4, one way ANOVA, **6F, G**). **(H–J)** Immunostaining images demonstrated CXCR2 (green) was predominantly co-localized with NeuN (red) as shown by overlapped staining (the rightmost panel, yellow). Scale bar, 100 μm.

Next, CXCR2 expression in the spinal cord after LQ injection was investigated. Immunohistochemical data showed that BCP-induced CXCR2 expression in the ipsilateral spinal dorsal horn on POD 12 when compared with sham controls (**Figures 6A–E**). The specificity of cxcr-2 antibody was confirmed by staining with first antibody preabsorbed with a blocking peptide with CXCR2-derived antigenic sequence (**Supplementary 1**). The daily intrathecal administration of LQ decreased CXCR2 immunofluorescence in the spinal dorsal horn *(F_4,15_ = 66.22, ^##^P < 0.01, ^###^P < 0.001, vs. BCP + Veh group; n = 4, one way ANOVA,* **Figure 6F**). Immunofluorescent data were further confirmed by WB analysis. Repeated LQ treatment down-regulated the expression of CXCR2 protein in the spinal cord of BCP rats *(F_4,15_ = 72.94, ^###^P < 0.001 vs. BCP + Veh group; n = 4, one way ANOVA,* **Figure 6G**). Intrathecal administration of 100 μg/kg LQ showed stronger effects on CXCR2 expression when compared with BCP-LQ (20 μg/kg) group *(F_4,15_ = 66.22, ^##^P < 0.01, ^###^P < 0.001, vs. BCP + Veh group; F_4,15_ = 72.94, ^###^P < 0.001 vs. BCP + Veh group; n = 4, one way ANOVA,* **Figures 6F, G**). These results suggested that intrathecal administration of LQ effectively inhibited CXCR2 upregulation in a dose-dependent manner. Double immunostaining of CXCR2/NeuN of walker 256 injection on 12 day animals indicated that CXCR2 was predominantly localized in spinal cord neurons (**Figures 6H–J**).

### LQ Alleviated Proinflammatory Cytokines Production in the Spinal Cord

**Figure 7:**
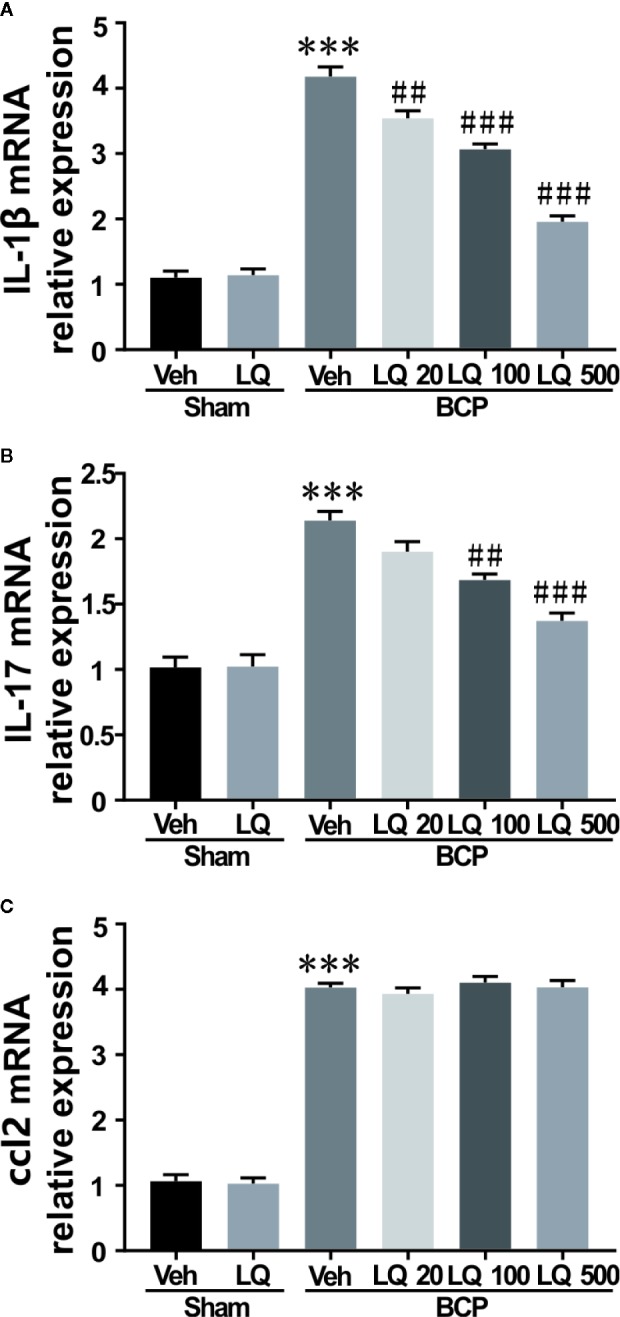
Effects of intrathecal injection of Liquiritin (LQ) on bone cancer pain (BCP)-induced upregulation of IL-1β, IL-17, and ccl2 levels in spinal dorsal horn were revealed by RT-PCR. **(A–C)** RT-PCR showed no remarkable differences regarding the levels of IL-1β, IL-17, and ccl2 between the sham + Veh group and sham + LQ 500 μg/kg group (F_5,18_ = 138.6, P > 0.05 vs. sham +Veh group; F_5,18_ = 41.14, P > 0.05 vs. sham +Veh group; F_5,18_ = 284.8, P > 0.05 vs. sham +Veh group; n = 4, one way ANOVA, **7A–C**). A significant upregulation of levels of IL-1β, and IL-17 as well as chemokine ccl2 were observed in BCP, and were increased by 3.80-, 2.11-, and 3.78-folds, respectively, on day 12 after injection of walker 256 cells (F_5,18_ = 138.6, ^***^P < 0.001 vs. sham +Veh group; F_5,18_ = 41.14, ^***^P < 0.001 vs. sham +Veh group; F_5,18_ = 284.8, ^***^P < 0.001 vs. sham +Veh group; n = 4, one way ANOVA, **7A–C**). Only IL-1β mRNA was decreased in the LQ 20 μg/kg group (F_5,18_ = 138.6, ^##^P < 0.01 vs. BCP + Veh group; n = 4, one way ANOVA, **7A**). **(A**, **B)** LQ 100 μg/kg decreased the expression of IL-1β and IL-17 mRNA to 73.41% and 78.83%, respectively, when compared with BCP group injected with vehicle (F_5,18_ = 138.6, ^###^P < 0.001 vs. BCP + Veh group, F_5,18_ = 41.14, ^##^P < 0.01 vs. BCP + Veh group; n = 4, one way ANOVA, **7A, B**). **(A**, **B)** Under the management of LQ 500 μg/kg, the expression of IL-1β and IL-17 mRNA was decreased to 46.83% and 64.21% (F_5,18_ = 138.6, ^###^P < 0.001 vs. BCP + Veh group, F_5,18_ = 41.14, ^###^P < 0.001 vs. BCP + Veh group; n = 4, one way ANOVA, **7A, B**). **(C)** No significant decrease was observed in the levels of ccl2 after the three concentrations of LQ administration following BCP surgery (F_5,18_ = 284.8, ***P < 0.001 vs. sham +Veh group, P > 0.05 vs. BCP + Veh group; n = 4, one way ANOVA, **7C**).

To evaluate the possible signal transduction pathways by which LQ alleviated BCP-induced hyperalgesia, the effect of intrathecal administration of LQ on the levels of pro-inflammatory cytokines such as IL-1β and IL-17 as well as chemokine ccl2 were investigated. LQ or vehicle was given daily for seven consecutive days and the spinal cord was dissected on day 12 after surgery. As shown in **Figures 7A, C**, no significant differences were observed in the levels of IL-1β, IL-17, and ccl2 between sham + Veh group and sham + LQ 500 μg/kg group *(F_5,18_ = 138.6, P > 0.05 vs. sham +Veh group; F_5,18_ = 41.14, P > 0.05 vs. sham +Veh group; F_5,18_ = 284.8, P > 0.05 vs. sham +Veh group; n = 4, one way ANOVA,* **Figures 7A–C**). RT-PCR results showed that the expression of IL-1β, IL-17, and ccl2 mRNA was increased by 3.80-, 2.11-, and 3.78-folds, respectively, on day 12 after injection of walker 256 cells (F_5,18_ = 138.6, ^***^P < 0.001 vs. sham +Veh group; F_5,18_ = 41.14, ^***^P < 0.001 vs. sham +Veh group; F_5,18_ = 284.8, ^***^P < 0.001 vs. sham +Veh group; n = 4, one way ANOVA, **Figures 7A–C**). LQ 500 μg/kg decreased the expression of IL-1β and IL-17 mRNA by 46.83% and 64.21%, respectively, when compared with BCP group injected with vehicle *(F_5,18_ = 138.6, ^###^P < 0.001 vs. BCP + Veh group, F_5,18_ = 41.14, ^###^P < 0.001 vs. BCP + Veh group; n = 4, one way ANOVA,* **Figures 7A, B**). By LQ 100 μg/kg, the expression of IL-1β and IL-17 mRNA was decreased to 73.41% and 78.83% *(F_5,18_ = 138.6, ^###^P < 0.001 vs. BCP + Veh group, F_5,18_ = 41.14, ^##^P < 0.01 vs. BCP + Veh group; n = 4, one way ANOVA,* **Figures 7A, B**). Only IL-1β mRNA showed a decrease in LQ 20 μg/kg group *(F_5,18_ = 138.6, ^##^P < 0.01 vs. sham +Veh group; F_5,18_ = 41.14, P > 0.05 vs. sham +Veh group; F_5,18_ = 284.8, P > 0.05 vs. sham +Veh group; n = 4, one way ANOVA,* **Figures 7A–C**). However, all the three dosages showed no obvious effects on the expression of ccl2 after BCP surgery *(F_5,18_ = 284.8, P > 0.05 vs. BCP + Veh group; n = 4, one way ANOVA,* **Figure 7C**).

### LQ Reduced LPS-Induced mRNA Increase of CXCL1 Expression in Astroglial-Enriched Cultures

**Figure 8:**
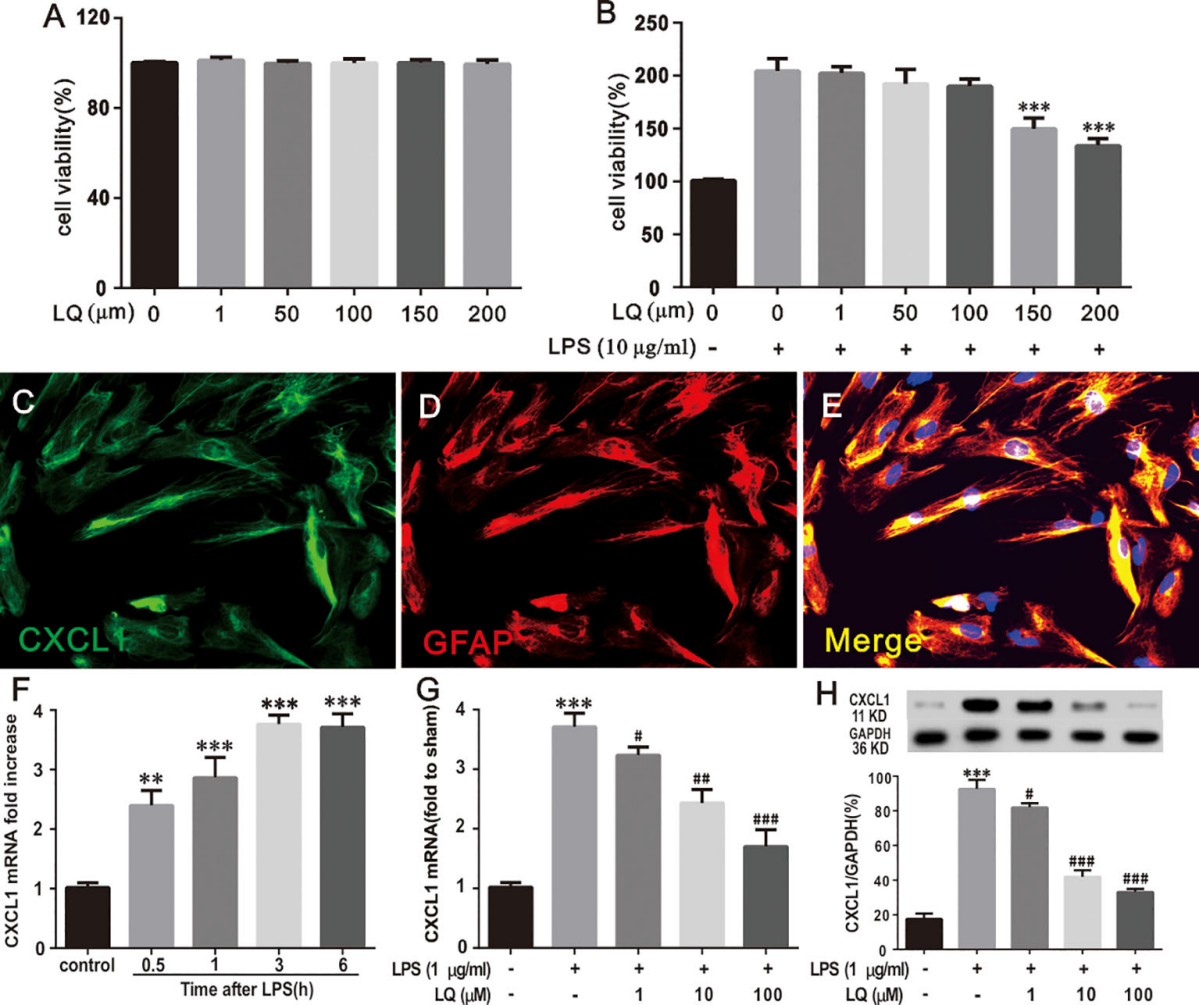
Liquiritin (LQ) reduces LPS-induced mRNA increase of CXCL1 expression in primary cultured astrocytes. **(A)** Astrocytes were treated with indicated concentrations (1, 50, 100, 150, and 200 μm) of LQ and assessed using MTT assay. The control cells were treated with DMSO (F_5,30_ = 0.1567, P > 0.05 vs. control; n = 6, one way ANOVA, **A**). **(B)** LPS stimulated astrocytes were exposed to LQ (1, 50, 100, 150, and 200 μm) and assessed using MTT assay (F_6,35_ = 19.61, ^***^P < 0.001 vs. control; n = 6, one way ANOVA, **8B**). **(C–E)** Double staining of CXCL1 with GFAP showed the CXCL1 expression by astrocytes. Scale bar = 50 μm **(F)** LPS (1 μg/ml) dramatically increased CXCL1 mRNA expression in primary astrocytes at 0.5, 1, 3 and 6 h (F_4,15_ = 24, ^**^P < 0.01,^***^P < 0.001 vs. control; n = 4, one way ANOVA, **F**). **(G, H)** LPS-induced CXCL1 upregulation was decreased by treatment with LQ (F_4,15_ = 25.2,^***^P < 0.001 vs. naïve, ^#^P < 0.05, ^##^P < 0.01,^###^P < 0.001 vs. control; F_4,15_ = 83.49, ^***^P < 0.001 vs. naïve, ^#^P < 0.05, ^###^P < 0.001 vs. control; n = 4, one way ANOVA, **8G, H**). All data are presented as means ± SEM.

To measure the cytotoxicity of LQ on astrocytes, the astrocytes were primarily cultured and then MTT assay was conducted. **Figure 8A** showed that the cell viability demonstrated no significant alterations during treatment with LQ for 24 h up to 200 μM concentration *(F_5,30_ = 0.1567, P > 0.05 vs. control; n = 6, one way ANOVA,* **Figure 8A**). Treatment with LQ (0–200 μM) followed by LPS (10 μg/ml) stimulation for 24 h showed acute toxicity from 150 μM (F_6,35_ = 19.61, ^***^P < 0.001 vs. control; n = 6, one way ANOVA, **Figure 8B**). Therefore, LQ concentration was fixed as 1, 50, and 100 μM in the subsequent experiments.

To verify whether LPS treatment, which induces pro-inflammatory cytokine expression, could directly reproduce pathological increase in CXCL1 expression in astrocytes, immunohistochemistry was conducted for CXCL1 and GFAP, and WB and RT-PCR were conducted for CXCL1 were performed. Double immunostaining of CXCL1/GFAP in astroglial-enriched cultures indicated that CXCL1 was localized in astrocytes (**Figures 8C–E**). LPS treatment (1 μg/ml) induced a significant increase in CXCL1 expression in astroglial-enriched cultures in a time-dependent manner (**Figure 8F**), (F_4,15_ = 24, ^**^P < 0.01,^***^P < 0.001 vs. control; n = 4, one way ANOVA, **Figure 8F**). The increase was obvious at 0.5 h and this was continued for more than 6 h. Notably, this increase was inhibited by LQ treatment for 24 h. LQ in a dose-dependent manner (1, 10, and 100 μM) suppressed LPS-induced CXCL1 release in astroglial-enriched cultures (**Figures 8G, H**), (F_4,15_ = 25.2, ^***^P < 0.001 vs. naïve, ^#^P < 0.05, ^##^P < 0.01,^###^P < 0.001 vs. control; F_4,15_ = 83.49, ^***^P < 0.001 vs. naïve, ^#^P < 0.05, ^###^P < 0.001 vs. control; n = 4, one way ANOVA, **Figure 8G, H**).

### LQ Indirectly Reduced CXCR2 Upregulation in Primary Neurons Mediated by Astroglial-Enriched-Conditioned Medium

**Figure 9:**
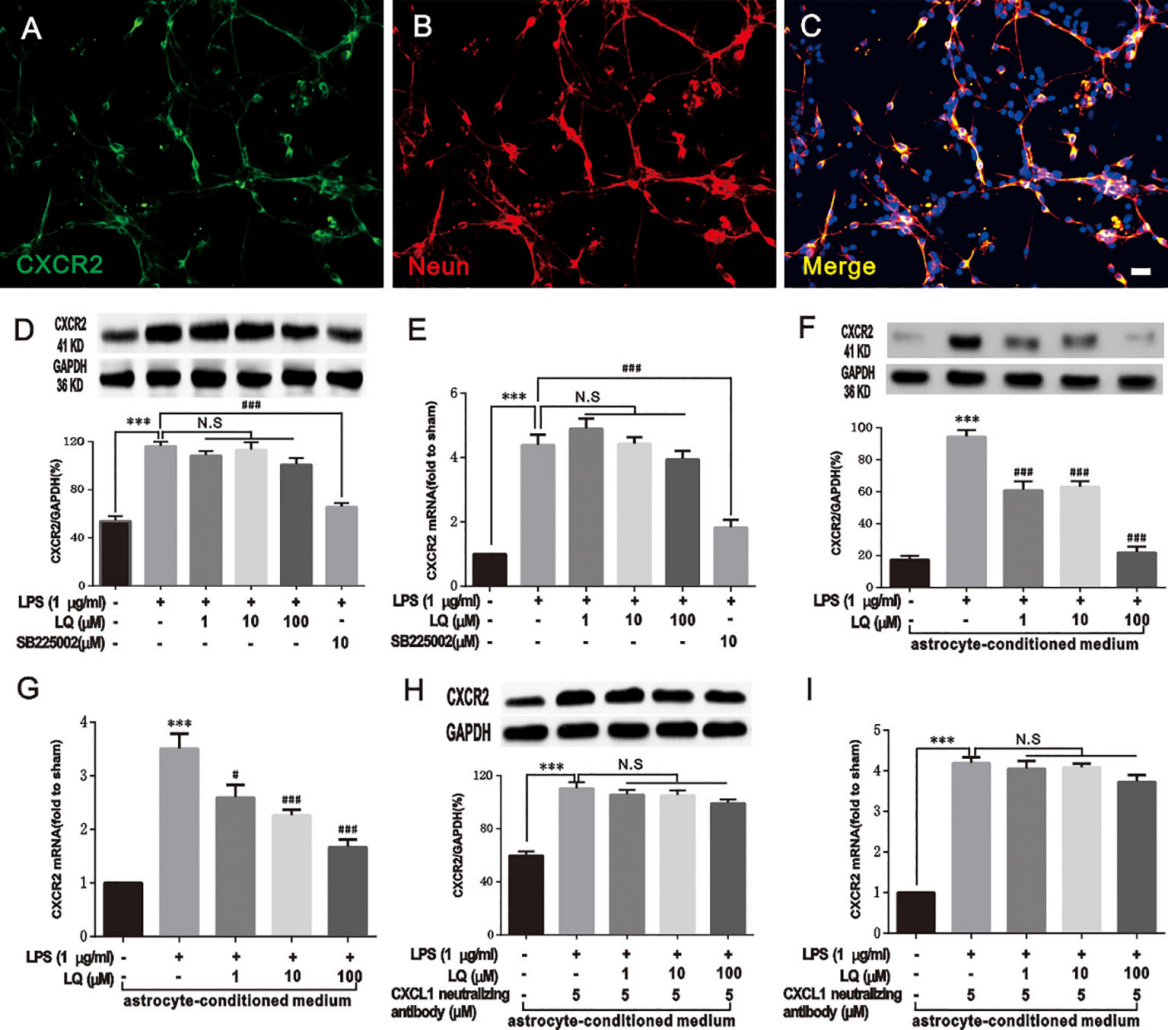
Liquiritin (LQ) indirectly reduces CXCR2 upregulation in primary neurons mediated by astrocyte-conditioned medium. **(A–C)** Double staining of CXCR2 with NeuN demonstrated the expression of CXCR2 by neurons. Scale bar = 50 mm. **(D**, **E)** LQ did not directly act on the increase in LPS-induced CXCR2 in primary neurons (F_5,18_ = 35.21, P > 0.05, ^***^P < 0.001, ^###^P < 0.001 vs. control, F_5,18_ = 40.77, P > 0.05, ^***^P < 0.001, ^###^P < 0.001 vs. control; n = 4, one way ANOVA, **D, E**). **(F**, **G)** LQ indirectly reduced the increase of CXCR2 mediated by astrocyte-conditioned medium (F_4,15_ = 63.6, ^***^P < 0.001 vs. Naive, ^###^P < 0.001 vs. control, F_4,15_ = 26.58, ^***^P < 0.001 vs. naïve, ^#^P < 0.05,^###^P < 0.001 vs. control; n = 4, one way ANOVA, **F, G**). **(H**, **I)** The astrocyte-conditioned medium incubated with LQ showed no effect on the increase of CXCR2 when CXCL1 was inhibited (F_4,15_ = 32.03, ^***^P < 0.001, P > 0.05 vs. control, F_4,15_ = 85.85,^***^P < 0.001, P > 0.05 vs. control; n = 4, one way ANOVA, **H, I**). All data are presented as means ± SEM. N.S., No statistical difference was found between two groups, P > 0.05.

Furthermore, the effect of LQ on CXCR2 in primary neurons was tested. The results of double immunostaining of CXCR2/NeuN in primary neurons indicated that CXCR2 was localized in neurons (**Figures 9A–C**). LPS treatment (1 μg/ml, 6 h) evoked a significant increase in CXCR2 expression in primary neurons. Our previous study demonstrated that LQ inhibited the expression of CXCR2 in the spinal cord (**Figures 6F, G**), (*F*_4,15_ = 63.6, ^###^ *P* < 0.001 vs. control group; *F*_4,15_ = 26.58, ^#^ *P* < 0.05, ^###^ *P* < 0.001 vs. control group; n = 4, one way ANOVA, **Figures 9F, G**). Interestingly, the up-regulation of CXCR2 expression by LPS treatment in primary neurons was reduced by SB225002 (10 mM), which is a selective CXCR2 antagonist, but not in all the 3 LQ groups (**Figures 9D, E**), (F_5,18_ = 35.21, P > 0.05, ^***^P < 0.001, ^###^P < 0.001 vs. control, F_5,18_ = 40.77, P > 0.05, ^***^P < 0.001, ^###^P < 0.001 vs. control; n = 4, one way ANOVA, **Figures 9D, E**). To verify whether treatment with astrocyte conditioned medium pre-treated with LQ that is substituted with half neuronal medium, WB and RT-PCR were performed for CXCR2, and the results showed that upregulation of neuronal CXCR2 expression has become sensitive to the effect of LQ (F_4,15_ = 63.6, ^***^P < 0.001 vs. Naive, ^###^P < 0.001 vs. control, F_4,15_ = 26.58, ^***^P < 0.001 vs. naïve, ^#^P < 0.05, ^###^P < 0.001 vs. control; n = 4, one way ANOVA, **Figures 9F, G**). These results suggested that LQ could indirectly reduce the increased CXCR2 expression mediated by astroglial-enriched-conditioned medium. Furthermore, LQ showed no effect on the increased CXCR2 expression when CXCL1 was inhibited and incubated with astrocyte-conditioned medium (**Figures 9H, I**), (F_4,15_ = 32.03, ^***^P < 0.001, P > 0.05 vs. control, F_4,15_ = 85.85, ^***^P < 0.001, P > 0.05 vs. control; n = 4, one way ANOVA, **Figures 9H, I**). These results further indicated the indirect effect of LQ in regulating CXCR2 expression.

## Discussion

Currently, BCP treatment severely influenced the quality of life of patients with bone metastasis, making it far from satisfactory. The present study examined the antinociceptive effects and possible underlying mechanisms of LQ in the BCP rat model. Firstly, the behavioral testing demonstrated that repeated administration of LQ alleviated mechanical allodynia of BCP rats. Secondly, LQ inhibited BCP-induced activation of astrocytes in the spinal cord. Thirdly, LQ effectively inhibited the activation of CXCL1/CXCR2 pathway and production of IL-1β and IL-17 in BCP rats. Finally, our results demonstrated that LQ treatment inhibited up-regulation of CXCL1 expression in astroglial-enriched cultures and indirectly acted on CXCR2 receptors in spinal cord neurons. Taken together, these results suggest that intrathecal LQ might attenuate BCP possibly by inhibiting the activation of astrocytes and production of proinflammatory cytokines and chemokines in the spinal cord. These findings demonstrated a new underlying mechanism and a new potential therapeutic target for BCP.

BCP involves a complex pain mechanism, exhibiting the elements of both inflammatory and neuropathic pain (Edwards and Mulvey, 2019). The BCP model of intramedullary injection of walker 256 cells is successfully established and is widely used to explore the mechanism of analgesic drugs due to its stable and sustaining pain-related behaviors (Akoury et al., 2019). In our present study, the mechanical withdrawal threshold showed a significant decrease on day 6 and continued to decline at a certain speed until day 18, which was in line with the previous research results (Hu et al., 2013; Ni et al., 2019a). LQ is one of the major constituents of Glycyrrhiza radix that exerts anti-inflammatory effect (Li et al., 2018), pro-apoptotic effect (Wei et al., 2017), neurotrophic effect (Chen et al., 2009), and anti-RA (Zhai et al., 2019). In addition, intragastric administration of LQ reduced hyperalgesia in a dose-dependent manner and inhibited the activation of glia (astrocyte and microglia) on CCI induced neuropathic pain (Zhang M. T. et al., 2017). In our present study, repeated intrathecal administration of LQ for seven consecutive days after injecting walker 256 cell reversed the established mechanical allodynia in rats, suggesting that LQ induced antinociception in BCP model and providing a direct evidence for the effect of LQ on the spinal cord. Interestingly, the analgesic effect of LQ at relatively higher doses was maintained till day 6 after drug withdrawal. Growing evidence also showed that LQ exerts considerable anti-tumor activities on various types of cancers (Zhou and Ho, 2014; He et al., 2017; Wei et al., 2017). These long-term analgesic and anti-tumor actions might make LQ as a potential therapeutic agent for BCP.

Spinal glial activation, mainly the microglia and astrocytes, is now considered as an important factor for the development and maintenance of allodynia and hyperalgesia in various chronic pain models, including inflammatory pain, neuropathic pain, and BCP (Cao et al., 2014; Ni et al., 2016; Jiang et al., 2017; Wang et al., 2018). The activated glial cells contributed to the early development and maintenance of chronic pain by releasing neuromodulators, such as proinflammatory cytokines and chemokines (Miyoshi et al., 2008; Liu et al., 2017). In the present study, BCP induced by intratibial inoculation of Walker 256 cells led to the activation of microglia and astrocytes in the spinal dorsal horn, which was consistent with that of the previous studies (Wang et al., 2011; Hu et al., 2012). The alleviation of mechanical allodynia after administration of LQ was also observed. Meanwhile, down-regulation of GFAP expression, but not Iba1, in the spinal dorsal horn was observed, indicating suppressed activity of only astrocytes and the proliferation of microglia remained unaffected by LQ. These data suggest that astrocyte signaling might act as suitable therapeutic targets of LQ for BCP.

The activated glial cells promoted pain transmission by releasing neuromodulators, such as chemokines and proinflammatory cytokines. The enhanced spinal neuroimmune and neuroinflammatory activities induce and maintain BCP (Shen et al., 2014; Lu et al., 2015; Liu et al., 2017). Mounting evidence suggests that chemokines and proinflammatory cytokines are mainly produced by glial cells in the spinal cord. For example, TNF-α and IL-6 are dominantly expressed in microglia (Berta et al., 2014; Rojewska et al., 2014, whereas CCL2, IL-17, and IL-1β are mainly expressed in the astrocytes of spinal cord (Zhang et al., 2005; Gao et al., 2009; Meng et al., 2013). We herein found that a high dose of LQ significantly decreased the expression of IL-1β and IL-17, suggesting the anti-inflammatory effect of LQ on astrocytes. Consistent with our results, LQ decreased the spinal IL-6, IL-1β, and TNF-α protein expression in chronic constriction injury induced neuropathic pain (Zhang M. T. et al., 2017).

Astrocyte response to LPS, a stimulator of inflammation, have been debated well for over two decades (Saura, 2007). LPS induced upregulation of proinflammatory cytokines in primary astrocytes (Ryu et al., 2019). In addition, LPS-induced proliferation was done through PI3K/AKT signaling pathway in normal human astrocytes (Zhang K. et al., 2017). LPS preconditioning also released HMGB-1 from reactive astrocytes (Xie et al., 2016), whereas recent scholars have stated that high purity of astrocytes culture did not respond to LPS (Liddelow et al., 2017). Various culture conditions (temperature or culture medium, etc) and sources of astrocytes (mouse or mice or human) can influence the results. For example, in highly purified astrocytes of mouse, LPS induced mostly an A1 (reactive astrocytes) predominant response, while human astrocytes did not (Tarassishin et al., 2014). The presence of microglia in astroglial-enriched cultures should not be ignored although there are certainly groups that used adequate methods to estimate and minimize the proportion of microglia (Saura, 2007). In our experiments we do not rule out the possibility of contamination with microglia cells. The presence of microglia in astroglial cultures in many cases is desirable as it allows the astroglial-microglial cross-talk that is extremely important in glial activation.

The results of MTT assay indicated that LQ has no effect on the viability of astroglial-enriched cultures. Further studies showed increased survival rate, which might be because of LPS-induced proliferation in astrocytes. LPS was also used to stimulate astrocytes and our results showed that LPS increased the expression of CXCL1 in astroglial-enriched cultures in a time-dependent manner. *In vitro* results revealed that LQ reduced inflammatory response in UVB-induced HACAT cells (Li et al., 2018). Here, we found that LQ suppressed the LPS-induced CXCL1 release in astroglial-enriched cultures in a dose-dependent manner. These data further supported the inhibitory role of LQ on the production of inflammatory mediators in the central nervous system.

Accumulating evidence indicated that immunemodulators play a key role in regulating synaptic plasticity and neuronal excitability, contributing to the persistence of various pain intensities (Jiang et al., 2017; Yang et al., 2017; Wu et al., 2019). The role of CXCL1, a member of the CXC family, depends on its primary receptor CXCR2 (Carreira et al., 2013). Spinal CXCL1 is produced by astrocytes and mediates pain *via* CXCR2 receptors in various chronic pain models (, 2014; Cao et al., 2014; Chen et al., 2014; Xu et al., 2014). Our previous study also found that CXCL1-CXCR2 signaling cascade played a role in glial-neuron interactions and in descending facilitation of BCP (Ni et al., 2019a). In agreement with these reports, the astrocytic CXCL1 and neural CXCR2 expression were increased in the spinal cord on day 12 after surgery. Additionally, the analgesic effects of LQ were mediated by downregulating CXCL1 and CXCR2. In *in vitro* conditions, LQ demonstrated no effect on the increase of CXCR2 in primary neurons, while it indirectly reduced the increase of CXCR2 mediated by astroglial enriched-conditioned medium. Interestingly, LQ showed no effect on the increase of CXCR2 when CXCL1 neutralizing antibody was used in astroglial enriched-conditioned medium, demonstrating an indirect effect of l-CDL in regulating CXCR2 expression.

In conclusion, LQ has a potent analgesic effect on BCP. Our results also indicated that the analgesic effect of LQ coincides with the inhibitory function of inflammatory reactions in SDH. Furthermore, the suppression of astrocytic CXCL1 and neural CXCR2 signaling cascade were involved in the analgesic effects of LQ. In astroglial-enriched cultures and primary neurons, CXCL1 was reduced by LQ in a dose-dependent manner, thus indirectly reducing the increase of CXCR2. These findings provided evidence for understanding the underlying mechanisms of anti-nociceptive effects of LQ in a BCP model and supported a novel strategy for treating BCP.

## Data Availability Statement

The datasets generated for this study are available on request to the corresponding author.

## Ethics Statement

The animal study was reviewed and approved by Animal Care and Use Committee of Jiaxing University.

## Author Contributions

HN carried out the animal surgery, behavioral testing, immunofluorescence and primary cell culture. MY participated in designing the experiments. MY conceived the project, coordinated and supervised the experiments. KX, YF, and MX participated in the animal surgery and behavioral testing. MX, HD, and QH participated in immunofluorescence. TW and SL participated in western blot experiments. JZ and LX participated in PCR experiments. HN and MX wrote the article. All authors read and approved the final manuscript.

## Funding

This study was supported by the National Natural Science Foundation of China (81901124), Natural Science Foundation of Zhejiang Province of China (LY20H090020, LGF20H090021, LQ19H090007), Medical and Health Science and Technology Research Program of Zhejiang Province (2020RC124, 2020RC122, 2019KY687), Science and Technology Project of Jiaxing City (2018AY32012), Emergency Science and Technology Special Fund of Jiaxing City (2020GZ30001), Construction Project of Anesthesiology Discipline Special Disease Center in Zhejiang North Region (201524 ), Key Discipline Established by Zhejiang Province and Jiaxing City Jointly--Pain Medicine (2019-ss-ttyx), Key Discipline of Anesthesiology of Jiaxing City (2019-zc-06) and Jiaxing Key Laboratory of Neurology and Pain Medicine.

## Conflict of Interest

The authors declare that the research was conducted in the absence of any commercial or financial relationships that could be construed as a potential conflict of interest.

## Supplementary Material

Supplementary 1 Antigenic peptide preabsorption blocks CXCR2 antibody staining. **(A, B)** Representative immunostaining images of naive rat spinal dorsal horn using CXCR2 antibody alone (control) or CXCR2 antibody+blocking peptide (Peptide preabsorption). **(C)** Summary of the fluorescence intensities of CXCR2 (control) or (Peptide preabsorption). (Scale bar, 100 μm.) ***P < 0.001. Student’s t test was used for the analysis.
