# Supplementary figures and images for: Liquiritin Alleviates Pain Through Inhibiting CXCL1/CXCR2 Signaling Pathway in Bone Cancer Pain Rat
Source: Front Pharmacol. 2020 Apr 24;11:436. doi: 10.3389/fphar.2020.00436 (PMC7193085; doi:10.3389/fphar.2020.00436)

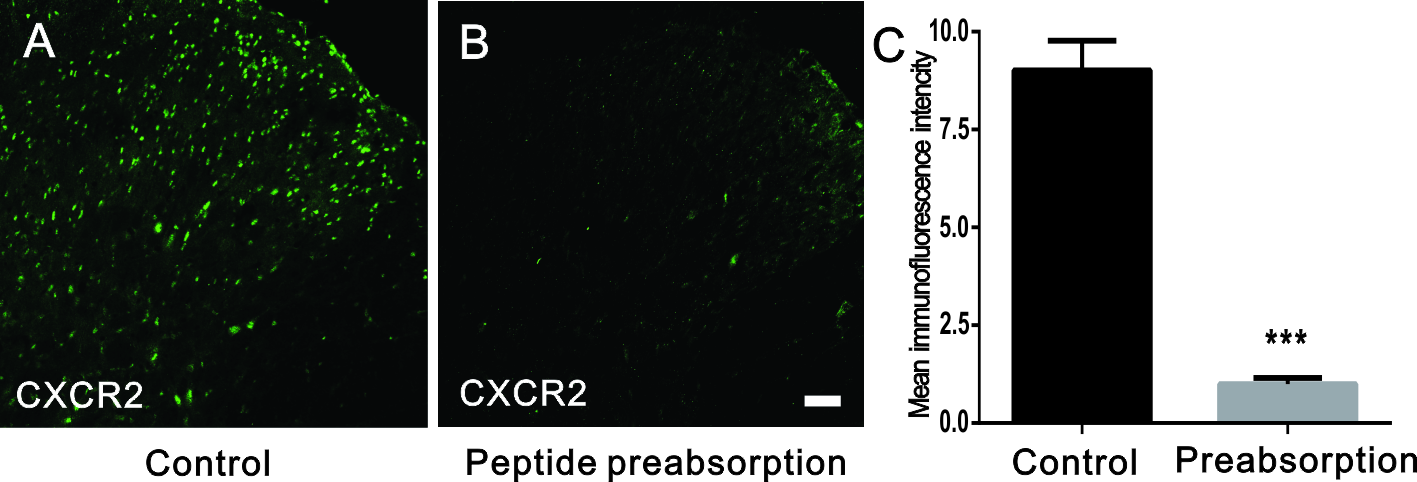

Supplement: Supplementary 1 — Antigenic peptide preabsorption blocks CXCR2 antibody staining. (A, B) Representative immunostaining images of naive rat spinal dorsal horn using CXCR2 antibody alone (control) or CXCR2 antibody+blocking peptide (Peptide preabsorption). (C) Summary of the fluorescence intensities of CXCR2 (control) or (Peptide preabsorption). (Scale bar, 100 μm.) ***P < 0.001. Student’s t test was used for the analysis. [file Image_1.tif]
